# Kre6 (yeast 1,6-β-transglycosylase) homolog, PhTGS, is essential for β-glucan synthesis in the haptophyte *Pleurochrysis haptonemofera*

**DOI:** 10.3389/fbioe.2023.1259587

**Published:** 2023-09-18

**Authors:** Mayuka Inukai, Naoya Kobayashi, Hirotoshi Endo, Koki Asakawa, Keisuke Amano, Yuki Yasuda, Ugo Cenci, Christophe Colleoni, Steven Ball, Shoko Fujiwara

**Affiliations:** ^1^ School of Life Sciences, Tokyo University of Pharmacy and Life Sciences, Hachioji, Japan; ^2^ National Institute of Technology, Tsuruoka College, Tsuruoka, Japan; ^3^ University of Lille, French National Centre for Scientific Research, UMR8576-UGSF-Unité de Glycobiologie Structurale et Fonctionnelle, Lille, France

**Keywords:** β-glucan, coccolithophorid, glucan synthase, Haptophyta, storage glucan, transglycosylase

## Abstract

Haptophytes synthesize unique β-glucans containing more β-1,6-linkages than β-1,3 linkages, as a storage polysaccharide. To understand the mechanism of the synthesis, we investigated the roles of Kre6 (yeast 1,6-β-transglycosylase) homologs, PhTGS, in the haptophyte *Pleurochrysis haptonemofera*. RNAi of *PhTGS* repressed β-glucan accumulation and simultaneously induced lipid production, suggesting that PhTGS is involved in β-glucan synthesis and that the knockdown leads to the alteration of the carbon metabolic flow. *PhTGS* was expressed more in light, where β-glucan was actively produced by photosynthesis, than in the dark. The crude extract of *E*. *coli* expressing PhKre6 demonstrated its activity to incorporate ^14^C-UDP-glucose into β-glucan of *P. haptonemofera*. These findings suggest that PhTGS functions in storage β-glucan synthesis specifically in light, probably by producing the β-1,6-branch.

## Introduction

Most organisms accumulate glucose polymers, glucans, as storage carbohydrates. Glucans are classified into α-glucans and β-glucans by the difference in the tertiary structure around anomeric carbon, carbon at the C_1_ site, in the glucose unit. Although many glucans have more than one type of linkage in a molecule, glucans with both α- and β-linkages are unknown. Although green lineage, including land plants and green algae, are known to synthesize starch, a mixture of α-1,4-linked and α-1,6-branched glucans, algae have a wider variety of glucans depending on the algal taxa (Ball et al., 2015). The primary algae, glaucophytes, rhodophytes, and chlorophytes, produce α-glucans. On the other hand, in the secondary algae, cryptophytes and dinoflagellates produce α-glucans, while ochrophytes, haptophytes, euglenophytes, and chlorarachniophytes produce β-glucans (Suzuki and Suziki, 2013).

Among β-glucans, the glucan of euglenophytes, paramylon, is stored in the form of granules surrounded by a single-layer membrane, unlike other β-glucans (Kiss and Triemer, 1988; Monfils et al., 2011). β-glucan of diatoms, chrysolaminarin, is a β-glucan consisting of a linear β-1,3-chain with β-1,6-branches stored in the vacuole (Chen et al., 2021). Haptophytes, *Emiliania huxleyi* and *Pleurochrysis* (*Chrysotila*) *haptonemofera*, have been reported to have β-glucans containing β-1,3- and β-1,6-linkages at a ratio of approximately 2:3 (Varum et al., 1986; Hirokawa et al., 2008).

**FIGURE 1:**
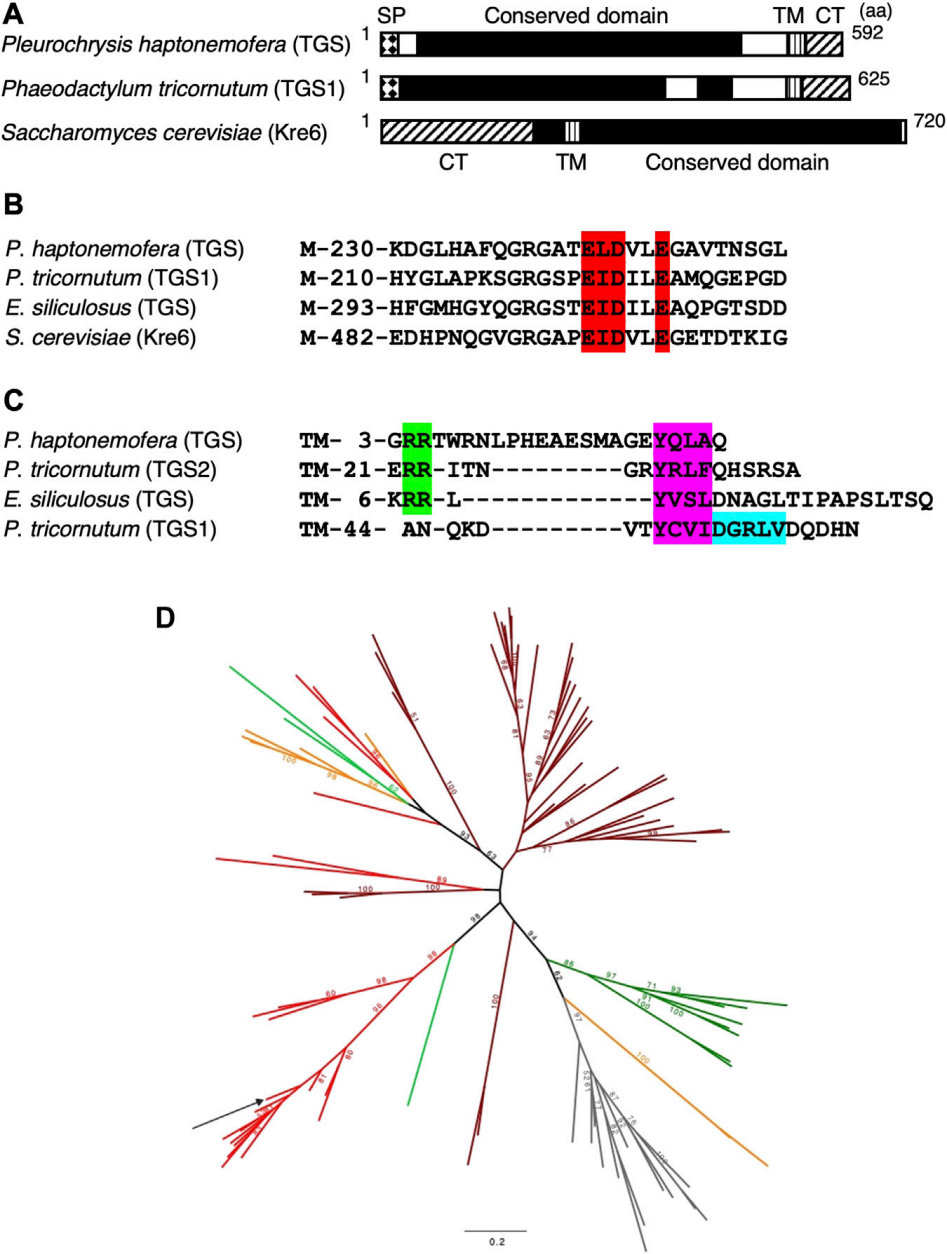
Predicted structures and phylogeny of Kre6 homologs (TGSs and GH16_2). **(A–C)** Domain structures **(A)**, β-glucan-binding site **(B)**, and sorting signals in the C-terminal region **(C)** of yeast Kre6 (SGD ID: S000006363) and its homologs from the haptophyte *P*. *haptonemofera*, the diatom *Phaeodactylum tricornutum* (JGI ID: TGS1 50238, TGS2 56509; Huang et al., 2016), and the brown alga *Ectocarpus siliculosus* (NCBI: CBJ31299). **(A)** SP, signal peptide; TM, transmembrane helix; CT, potential cytoplasmic tail. **(B)** Red: β-glucan-binding site; M, methionine. **(C)** Green: di-basic motif RR; purple: Tyr-based motif YXXΦ; blue: di-Leu-type motif DXXLL (Huang et al., 2016); TM, transmembrane helix. **(D)** Phylogenetic tree of the GH16_2 β-glucan branching enzyme. The unrooted maximum likelihood-based tree was obtained with the IQ-TREE under the LG4X model. Bootstraps from 100 repetitions are mapped onto the branch, with only bootstrap values > 50% shown. The scale bar shows the inferred number of amino acid substitutions per site. Sequences are highlighted in brown for Stramenopiles, red for Haptophyta, light green for Rhizaria/Cercozoa, dark green for Chloroplastida, orange for alveolata, and gray for fungi (Supplementary Figure S1). The sequence from *P. haptonemofera* is indicated with an arrow. The GH16_2 sequences are present in various organisms, and if their enzymological function is well-conserved, they may be involved in either storage or cell wall polysaccharide metabolism. The sequence of *P. haptonemofera* belongs to a highly supported monophyletic group of Haptophyceae (Haptophyta) GH16-2 enzymes likely to be all similarly involved in the synthesis of branched storage β-glucan polysaccharides as no β-glucan cell wall has been described in this lineage to the best of our knowledge.

The yeast cell wall consists of mannan protein (50%), β-1,3-glucan (40%), β-1,6-glucan (10%), and chitin (1%–3%). These constituents are synthesized through distinct pathways, but the β-1,6-glucan-synthesizing pathway has not been fully elucidated. The K1-killer yeast exotoxin can kill sensitive yeast by forming channels in the plasma membrane, and during the process, β-glucan is necessary as a receptor. A series of genes involved in β-1,6-glucan synthesis have been isolated from K1-killer resistant (*kre*) mutants, utilizing the characteristic that mutants with less β-glucan show K1-killer resistance (Brown et al., 1993). One of the gene products is Kre6, which is a type II membrane protein having one transmembrane domain and directing its N-terminal to the cytosol (Figure 1A). It has a glycoside hydrolase family 16 (GH16) domain on the C-terminal side, suggesting its activity for glucan linkages or elongation (Montijn et al., 1999). A Kre6-deficient mutant displays much less β-glucan contents than the wild type, and a double disruptant of Kre6 and its homolog Skn1 are lethal, indicating that both proteins are essential for β-1,6-glucan synthesis (Roemer et al., 1993). It is also reported that incubation of permeabilized yeast cells with UDP-glucose produces a polymer chemically identical to the β-1,6-glucan isolated from the cell wall (Aimanianda et al., 2009). Currently, it seems more difficult to detect β-1,6-glucan synthesis than β-1,3-glucan production. For β-1,3-glucans, the pathway and proteins involved in the biosynthesis have been almost completely clarified, while for β-1,6-glucans, the mechanism of biosynthesis is not known at all, although several involved proteins have been identified (Lesage and Bussey, 2006). For β-1,3-glucans, membrane proteins that play an important role in synthesizing the glucan and exporting it to the outside of the cell membrane have been identified, while for β-1,6-glucans, such a protein with several transmembrane regions and a catalytic domain has not been identified. This may be related to the research delay.

In microalgae, enzymes involved in the β-glucan metabolism pathway have been studied mainly in euglenophytes and diatoms (Chen et al., 2021; Huang et al., 2023). In *Euglena gracilis*, a paramylon membrane fraction including glucan synthase-like 2 (EgGSL2) was prepared, and the elongation activity of the β-1,3-glucan was detected after incubation with UDP-glucose (Bäumer et al., 2001; Tanaka et al., 2017). In the diatom *Phaeodactylum tricornutum*, two Kre6 homologs, 1,6-β-transglycosylases (TGS1 and TGS2) were found, and their localization in the vacuole and complementation of Kre6-deficient yeast mutants suggested that both are involved in β-1,6-branch synthesis (Huang et al., 2016). Studies on β-glucans of microalgae are exclusively focused on β-1,3-glucans (Chen et al., 2021). For β-1,6-glucans, most reports indicate indirect evidence that shows a decrease in the cell wall and/or β-glucan in gene-deficient mutants, and few studies directly monitor synthetic activity.

Haptophytes are considered secondary algal groups, which evolved after the secondary endosymbiosis of a rhodophyte organism into a heterotrophic organism (Edvardsen et al., 2000). The fact that rhodophytes synthesize α-glucans while haptophytes produce β-glucans suggests that this ability of haptophytes might be deduced from the heterotrophic host. Although many β-glucans have β-1,3-chains as their main chains, haptophytes, including *P*. *haptonemofera*, have unique β-glucans that have more β-1,6-linkages than β-1,3-linkages (Hirokawa et al., 2008). Knowledge about β-glucans in the haptophytes is expected to be valuable for understanding endosymbiosis and evolution. In the haptophyte *P*. *haptonemofera*, the structure of β-glucans and their degrading enzyme activity have been characterized, but the β-glucan-synthesizing enzyme has not been identified yet.

Homology search of candidate genes using the *Pleurochrysis* transcript sequence database Pleurochrysome (Yamamoto et al., 2016) has revealed that the yeast *Kre6* homolog is expressed in *P*. *haptonemofera*. In this study, we investigated the effect of its knockdown on the β-glucan amount and its gene expression in the haptophyte *P*. *haptonemofera*, which synthesizes unique β-glucans possessing more β-1,6 linkages than β-1,3-linkages. Furthermore, the UDP-glucose- and β-glucan-reacting activities of the recombinant protein were demonstrated *in vitro*, although the branching activity has not been experimentally demonstrated yet.

## Materials and methods

### Algal cells and culture conditions

Coccolith-bearing cells of *Pleurochrysis* (*Chrysotila*) *haptonemofera* were grown photoautotrophically, as described previously (Fujiwara et al., 2007). The cells were cultured in Daigo’s IMK medium (Nihon Pharmaceuticals, Tokyo, Japan) or artificial seawater-based Eppley’s medium (SLEP; Endo et al., 2016) at 20°C under continuous illumination at 40 μmol photon m^−2^ s^−1^ with constant bubbling of air.

### Determination of the *Kre6* homolog (*PhTGS*) mRNA sequence and prediction of the PhTGS domain structure

A full-length *PhTGS* mRNA sequence was obtained by 5′-RACE and RT-PCR using primers designed based on a *Kre6* homolog sequence that was found in the EST database Pleurochrysome (Yamamoto et al., 2016) and in *de novo* assembled sequences from RNA-seq data. Total RNA of *P*. *haptonemofera* was prepared using Isogen with a Spin Column (Nippon Gene, Japan), and then, cDNA synthesis and 5′-RACE were performed using the SMARTer^®^ RACE 5′/3′Kit (TaKaRa Bio, Japan). For 5′-RACE, a nested PCR was carried out, using PhTGS-5′RACE-1 and PhTGS-5′RACE-2 (Supplementary Table S1) as gene-specific primers for the first and second rounds of PCRs, respectively, and the PCR products were cloned in the plasmid pRACE. For the confirmation of the internal sequence, the coding region was amplified using primers PhTGS-pET-F and PhTGS-pET-R (Supplementary Table S1), and the PCR products were inserted into pET-15b (Novagen) by In-Fusion cloning (TaKaRa Bio). The insert in the obtained plasmid pET-15b-PhTGS was sequenced, as well as those of the 5′-RACE products (DDBJ; accession number, LC773529).

Searches for the signal peptide and transit peptide were conducted using SignalP 4.1 and ChloroP 1.1, respectively. For the search for the transit peptide, the sequence from which the signal peptide was removed was applied. A domain search was performed by NCBI’s Conserved Domain Search. The membrane protein and transmembrane helix were predicted using ExPASy ProtScale and SOSUI and TMHMM (v. 2.0) servers, respectively. InterPro was utilized to search the protein family membership, domain, homologous superfamily, and GO terms. WoLF PSORT was used for the prediction of cellular localization.

### Phylogenetic analysis

Sequences already annotated in the previous study were taken, as well as sequences from *P. haptonemofera*; we subsequently enriched using homologous research with BlastP against sequences of the non-redundant protein sequence database of the NCBI and sequences from other databases (MMETSP and data publicly available). We then aligned the sequences using MUSCLE (Edgar, 2004); block selection was carried out using BMGE with a block size of 4 and the BLOSUM30 similarity matrix. Trees were generated using IQ-TREE with 100 bootstrap repetitions using the LG4X model.

### RNAi of PhTGS in *Pleurochrysis*

Three kinds of double-stranded RNA (dsRNA) were transferred into *P*. *haptonemofera* protoplasts by the method developed by Endo et al. (2016). For dsRNA preparation, DNA having the T7 RNA polymerase consensus binding site at the 5′-terminus was synthesized by PCR using PhTGS-ND-1F and-1R (for targeting 5′ half of the CDS), PhTGS-ND-2F and -2R (for targeting 3′ half of the CDS), and PhTGS-ND-3F and -3R (for targeting the region around 5′ end of the CDS) as primer pairs (Supplementary Table S1), and then, *in vitro* transcription was performed by T7 RNA polymerase (TaKaRa Bio) using the DNA fragment as a template. The synthesized RNA molecules with complementary sequences were annealed to synthesize dsRNA. *P. haptonemofera* protoplasts were prepared using the hypoosmotic K^+^ treatment method (Takayanagi et al., 2007) modified by Endo et al. (2016). The three kinds of dsRNA and control dsRNA (GFP dsRNA) were transferred into the protoplasts by the PEG-mediated method (Endo et al., 2016), and the dsRNA-introduced cultures were analyzed as KD-tgs_1, KD-tgs_2, KD-tgs_3, and control, respectively.

### Measurement of the *PhTSG* mRNA level by quantitative RT-PCR (qRT-PCR)

Total RNA isolated from cells at 3 days after RNAi using Isogen (Nippon Gene) was treated using the TURBO DNA-free™ Kit (Ambion, United States), and then reverse-transcribed using the QuantiTect Reverse Transcription Kit (QIAGEN, Germany). qPCR was performed using the Rotor-Gene SYBR^®^ Green PCR Kit (QIAGEN) using a Rotor-Gene Q Real-time PCR System (QIAGEN) and primer sets PhTGS-qPCR-F and -R and Actin-qPCR-F and -R. The PCR thermal profiles comprised 45 cycles of 95°C for 5 s and 60°C for 10 s.

### Determination of the β-glucan

Small-scale β-glucans were measured according to Hirokawa et al. (2008)’s method with a slight modification. Briefly, cells in 2 mL of cultures at 3 days after RNAi were harvested by centrifugation (18,500 × g) and then suspended in 300 μL of the McIlvain buffer (pH6.0). After sonication, methanol was added to the suspension, and the mixture was incubated at 4°C for more than 4 h. After centrifugation, the β-glucan-containing pellet was dried up and then solved in 25 μL of the McIlvain buffer. The β-glucan was converted to glucose by treatment using westase (TaKaRa Bio), and the total glucose content was determined using hexokinase (Oriental Yeast Co., Ltd, Japan) and glucose-6-phosphate dehydrogenase (Roche Diagnostics K.K., Germany), according to Bergmeyer et al. (1974). Westase is an enzyme cocktail, including β-1,6-glucanase and β-1,3-glucanase, for yeast protoplast preparation, and it is demonstrated to completely degrade the β-glucan of *P. haptonemofera* into glucose (Hirokawa et al., 2008).

### Lipid detection by BODIPY staining

Approximately 0.5 μL of BODIPY 493/503 (Life Technologies, United States) stock solution (1 mg/mL DCMU) was added to 100 μL of culture. After mixing and incubation for a couple of minutes, cellular fluorescence was detected via fluorescence microscopy (BX53 using the NIBA filter, Olympus, Japan) and flow cytometry (SH800, Sony, Japan). For flow cytometry, the fluorescence of BODIPY 493/503 and that of chlorophyll was detected using FL2 and FL4 detectors with filter sets for FITC (518 nm) and 7AAD (647 nm), respectively. Signals from 5,000 particles that were gated using FL4-area were collected using a logarithmic scale, according to a previous report (Takayanagi et al., 2007).

### Expression of PhTGS in *E. coli*

For the induction of the recombinant protein expression, logarithmic-phase cells of *E*. *coli BL21* (DE3) carrying pET-15b-PhTGS were further cultivated in the presence of 0.5 mM IPTG at 20°C for 16 h. The cells collected and resuspended in PBS without KCl were disrupted by sonication using a Sonifier 250D instrument (Branson, United States), and then, a soluble fraction was obtained by centrifugation (20,000 g for 10 min). The soluble and insoluble fractions were applied to a 10% SDS-PAGE gel, and the proteins were visualized using Coomassie Brilliant blue R-250 stain (CBB).

### Detection of activity of β-glucan synthesis

Using the soluble fraction of rPhTGS-expressing *E*. *coli*, β-glucan-synthesizing activity was measured. Approximately 1.3 mg/mL of the protein-containing soluble fraction was incubated at 20°C for up to 24 h in a total of 100 μL of a reaction solution [0.01 mM DTT, 0.2 mg of *P*. *haptonemofera* β-glucan (Hirokawa et al., 2008), 3.2 mM UDP-glucose, and 0.75 nmol [U-^14^C] UDP-glucose (1.2 × 10^10^ Bq mmol^−1^)]. The reaction was stopped by adding 2 mL of 70% ethanol. The precipitate was filtered using a glass-fiber filter (Whatman GF/C), rinsed with 15 mL of 70% ethanol, and dried at room temperature. The incorporated ^14^C was counted using a liquid scintillation counter. Protein concentrations were determined according to the method of Bradford (1976) with BSA as the standard, using the Coomassie protein assay reagent (Pierce Chemicals, United States).

## Results and discussion

### Domain structure and phylogeny of TGSs

Based on the *Kre6* homolog sequence found both in the EST database Pleurochrysome (Yamamoto et al., 2016) and in *de novo* assembly sequences of RNA-seq, 5′-RACE and RT-PCR were performed, and a full-length RNA sequence was determined. The gene product was deduced to be a 64-kDa protein with a pI of 5.11. The protein was predicted to have a signal peptide of 22 amino acids but not a transit peptide to the plastid (Figure 1A). The domain search showed a conserved domain in the center of the protein [SKN1 domain, 48–461 aa; β-glucan-binding site, 245–250 aa (Figure 1B)], followed by a transmembrane region and a cytosolic tail. This structure is very similar to TGS1 and TGS2 of the diatom *Phaeodactylum tricornum* but different from that of yeast Kre6 (Huang et al., 2016). Thus, the Kre6 homolog in *P*. *haptonemofera* was designated as PhTGS. The C-terminal structure is not homologous compared to those of ochrophytes, but two types of sorting motifs necessary for transport to the trans-Golgi network (Hanton et al., 2005; Hwang and Robinson, 2009; Robinson and Pimpl, 2014), the di-basic motif RR and the Tyr-based motif YXXΦ, are found in PhTGS, as in *P*. *tricornutum* TGS2 and *Ectocarpus siliculosus* TGS (Figure 1C; Huang et al., 2016), suggesting its transport to the vacuole or the cytoplasmic membrane via the trans-Golgi network. In addition, WoLF PSORT software predicted that PhTGS is localized in the vacuole (vacuole: 7, extracellular: 3, chloroplast: 2, ER: 2). Thus, the protein is probably localized in the vacuole as in ochrophytes (Huang et al., 2016).

The molecular phylogenetic tree of GH16_2 suggests that haptophyte TGSs form a sister clade to stramenopile TGSs, and the clade consisting of haptophytes and stramenopiles TGSs is sister to the cell wall-forming GH16_2s of fungi and Chlorophyta (Figure 1D). Haptophytes maintaining only β-storage glucan metabolism might be evolved from a common ancestor of haptophytes and stramenopiles possessing both α- and β-storage glucan metabolism, as in Gyrista, consisting of Oomycota and Ochrophyta (Chabi et al., 2021).

### Effects of RNAi of PhTGS on the β-glucan amount

**FIGURE 2:**
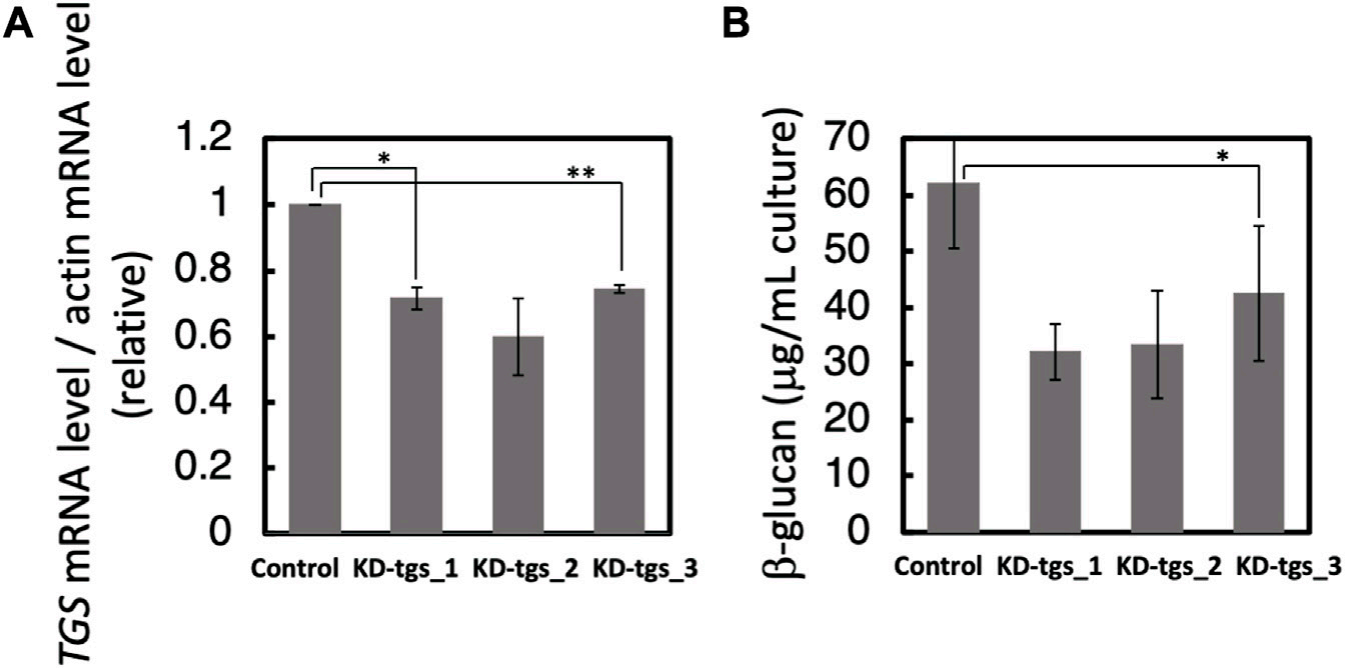
Effects of knockdown of *PhTGS* on the amount of β-glucan. **(A)** Knockdown confirmation of *PhTGS* expression at 3 days after RNAi. The PhTGS mRNA level was determined by qRT-PCR using total RNA from each RNAi line. The actin mRNA level was also measured as a control. **(B)** The cellular β-glucan amount at 3 days after RNAi. The bars represent means ± SE (*n* = 3) (Student’s t-test, *p < 0.05, and **p < 0.01).

To know whether PhTGS is actually involved in β-glucan synthesis, we performed a knockdown of PhTGS by RNAi. Three kinds of double-stranded RNA for targeting to the 5′ half, 3′ half, and around 5’ end regions of the CDS were prepared and introduced to *P*. *haptonemofera* protoplasts by the PEG method to obtain reliable results. The *PhTGS* mRNA level was decreased by around 30%–50%, when compared to the control (GFP dsRNA) (Figure 2A). At the same time, the cellular β-glucan amount was decreased by approximately 30%–50% (Figure 2B). Significant differences were detected between control and KD-tgs_1 and KD-tgs_3 in 2a, and KD-tgs_3 in 2b (the *p*-values were <0.05), although differences between the control and KD-tgs_2 in 2a and KD-tgs_1 and KD-tgs_2 in 2b were not significant (the *p*-values were 0.051∼0.076). These relatively high *p*-values may be due to the high SEs in the RNAi experiments. On the other hand, it was clearly shown that there was no significant difference among the knocked-down cells tested (the *p*-values among KD-tgs_1, _2, and _3 were 0.3∼0.9). These findings suggest that PhTGS plays an essential role in β-glucan synthesis.

### Effects of RNAi of PhTGS on the lipid content

**FIGURE 3:**
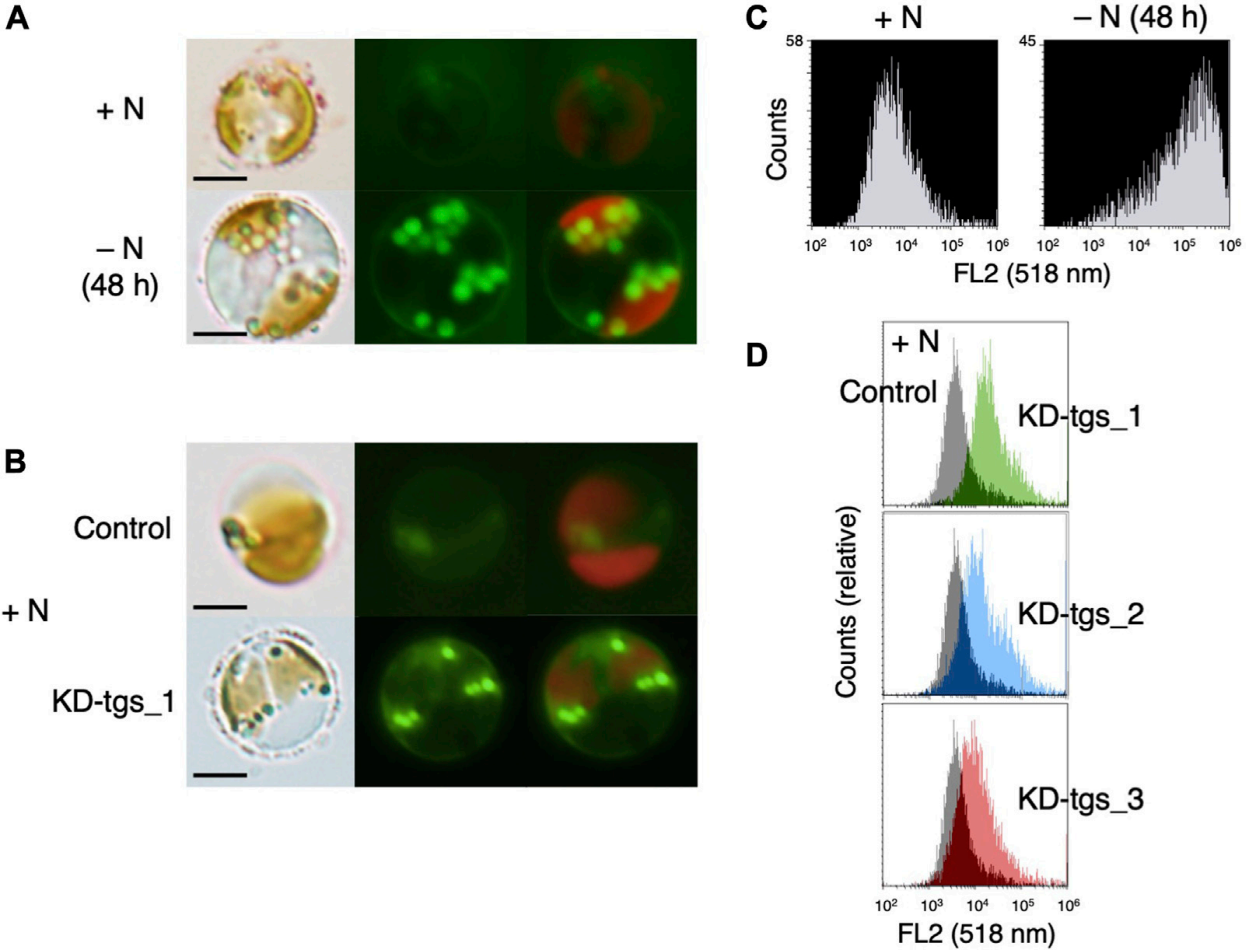
Lipid accumulation under N-depleted **(A, C)** or *PhTGS* knockdown **(B, D)** conditions. **(A, C)** Cells cultivated under N-replete and N-depleted conditions were stained with BODIPY, and cellular fluorescence was observed via fluorescent microscopy **(A)** and flow cytometry **(C)**. **(B, D)** Cells which had been grown under N-replete conditions for 72 h after treatment with three kinds of dsRNA for targeting to PhTGS (KD-tgs_1, _2, and _3) and control dsRNA (control) were stained with BODIPY, and cellular fluorescence was detected via fluorescent microscopy **(B)** and flow cytometry **(D)**. Lipid bodies were observed by fluorescent microscopy **(A, B)**, and the amount of oil droplets in the algae was analyzed by flow cytometry **(C, D)**. Scale bars = 5 μm.

In α-glucan-accumulating microalgae and β-glucan-containing diatoms, it is reported that an impaired storage glucan synthesis leads to an increase in the lipid content and lipid body (Siaut et al., 2011; Daboussi et al., 2014; Hildebrand et al., 2017; Chen et al., 2021). Thus, to determine whether a similar phenomenon is observed in the β-glucan containing haptophytes, we observed PhTGS knocked-down cells after BODIPY staining that stains the lipid body. Fluorescence microscopy indicated that the lipid body increased in PhTGS knocked-down cells even under N-replete conditions, although the number of lipid bodies is less compared to those in normal cells under N-starved conditions (Figures 3A, B). To confirm this observation, we performed the flow cytometry of the cells (Figures 3C, D). The fluorescence of BODIPY 493/503 and that of chlorophyll were detected using FL2 (518 nm) and FL4 (647 nm) detectors, respectively, and signals from cells that were gated by FL4 were collected on a logarithmic scale. The flow cytometry demonstrated that the amount of oil droplets in the cell increased in PhTGS knocked-down cells even under N-replete conditions, although the amount of oil droplets is less compared to those in normal cells under N-starved conditions (Figures 3C, D), similarly to fluorescent microscopy (Figures 3A, B). Significant differences were not observed among the three kinds of double-stranded RNA (Figure 3D). These findings suggest that the carbon metabolic flow is largely changed between glucans and lipids depending on their synthetic activities and C/N balance in β-glucan-accumulating haptophytes, as in α-glucan-accumulating microalgae (Siaut et al., 2011) and β-glucan-accumulating diatoms (Daboussi et al., 2014; Hildebrand et al., 2017; Chen et al., 2021).

### Expression of the *PhTGS* gene in *P*. *haptonemofera*

**FIGURE 4:**
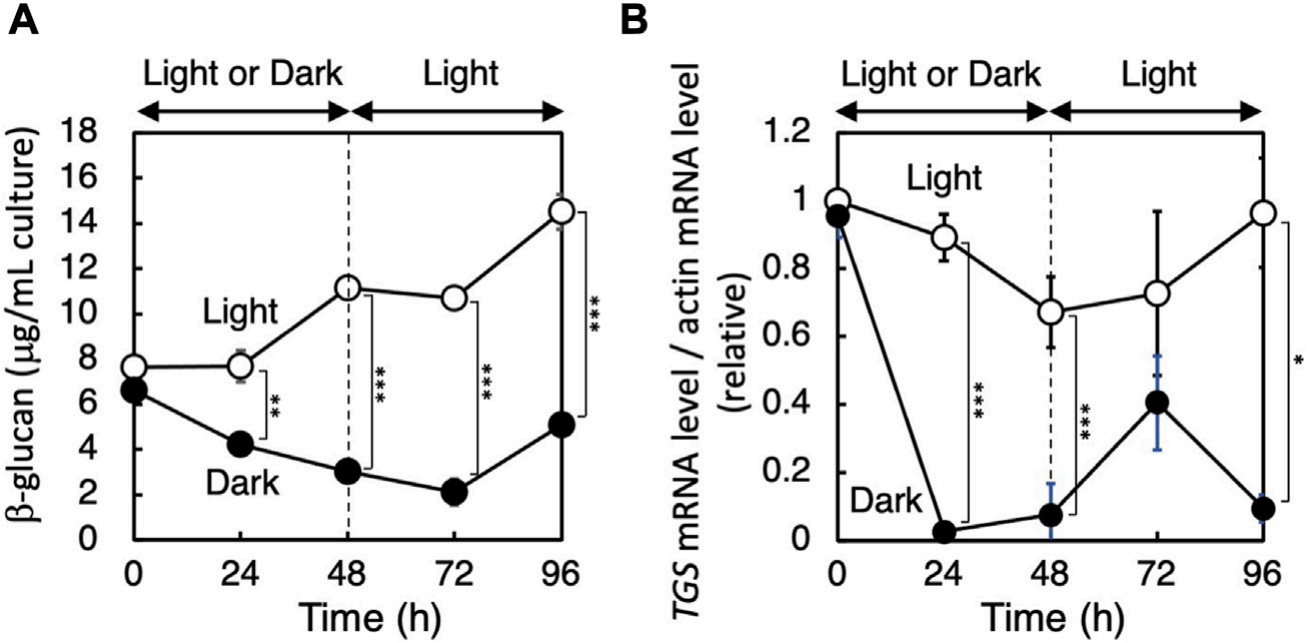
Effect of light on the amount of β-glucan **(A)** and expression of the *PhTGS* gene **(B)**. Cells grown until the logarithmic phase in light were transferred to dark or light conditions for 48 h and then back to light conditions. The *PhTGS* and actin mRNA levels were measured by qRT-PCR, and the ratio is plotted relative to those at 0 h. The bars represent means ± SD (*n* = 3) (Student’s t-test, *p < 0.05, **p < 0.01, and ***p < 0.001).

To know if the expression pattern is related to β-glucan synthesis, we investigated the effect of light on the *PhTGS* mRNA level. Logarithmic-phase cells that were actively synthesizing β-glucans were used for the experiments. The amount of β-glucans steadily increased in the cultures under continuous light, while it gradually dropped when cultures were placed in the dark (Figure 4A). Meanwhile, the *PhTGS* mRNA level was not significantly changed in the light, while it drastically decreased in the dark (Figure 4B). Taken together, the co-occurrence of active *PhTGS* gene transcription and β-glucan biosynthesis in the presence of light strongly suggests that PhTGS activity plays a role in the β-glucan anabolic path of *P. haptonemofera*, as in the diatom *P. tricornutum*. In *P*. *tricornutum*, genes involved in the β-glucan biosynthesis, *PtUGP2*, *PtBGS*, and P*tTGS1* have a coordinated diel expression pattern, with an increase at the beginning and decrease at the end of the light period (Skogen et al., 2013).

### β-glucan synthesis activity of PhTGS expressed in *E. coli*

**FIGURE 5:**
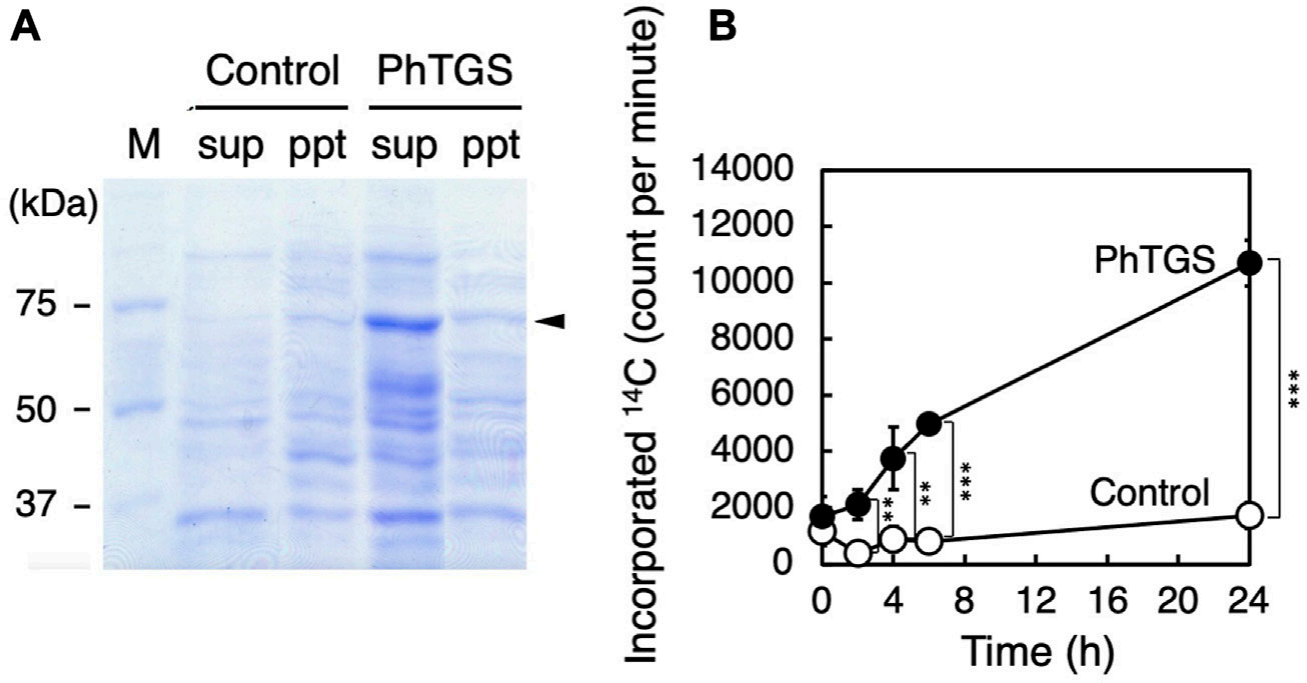
TGS activity by recombinant PhTGS. **(A)** SDS-PAGE of PhTGS expressed in *E. coli* BL21 (DE3). The expression was induced in the presence of IPTG, the cells were disrupted by sonication, and then the suspension was fractionated by centrifugation. **(B)** Incorporation of ^14^C-UDP-glucose into β-glucans by the supernatant fraction including recombinant PhTGS. As a control, an empty vector-carrying *E. coli* was also analyzed under the same conditions as those for PhTGS. Each assay mixture included 1.3 mg/mL of proteins. The bars represent means ± SD (*n* = 3) (Student’s *t*-test, *p < 0.05, **p < 0.01, and ***p < 0.001).

To understand the enzyme activity of PhTGS, we carried out the *in vitro* assay using the recombinant protein. The full-length CDS of PhTGS was inserted into an expression vector pET-15b, and an *E*. *coli* strain BL21 (DE3) was transformed with the plasmid. Expression of PhTGS in the transformant was induced, as described in Materials and Methods. The desired protein with a molecular mass of about 66 kDa (the His-tag including up- and downstream region, 2 kDa + PhTGS, 64 kDa) was detected as a band of approximately 70 kDa in the soluble fraction by SDS-PAGE (Figure 5A). The slightly smaller mobility on SDS-PAGE gels may be due to a positive charge of the His-tag.

The glucan synthase activity was measured in the soluble protein fraction at 20°C, using ^14^C-UDP-glucose as a substrate and β-glucan of *P*. *haptonemofera* as a primer. ^14^C-UDP-glucose was used because its incorporation into β-glucans was detectable with very high sensitivity. Specifically, the reaction was stopped by adding ethanol at appropriate times, and the ^14^C-glucose incorporated into the precipitate, including β-glucans, was counted in a liquid scintillation counter. The incorporation of the ^14^C-glucose moiety of UDP-glucose into β-glucans was significantly increased when compared with the soluble protein fraction of an empty vector-carrying *E*. *coli* (control) (Figure 5B). This finding suggests that PhTGS has an activity for the elongation of β-glucans using UDP-glucose. Furthermore, this fact infers that β-glucan-synthesizing organisms including oomycetes and ochrophytes might also have a glucan-branching mechanism utilizing TGS, which is different from that of α-glucan-containing organisms, where branching enzymes catalyze the formation of branch points in α-glucans by cleaving α-1,4-glucosidic linkages and then transfer the released oligosaccharide chains to C-6 hydroxyl groups. To confirm this hypothesis, further experiments for the clarification of the PhTGS reaction mechanism are necessary, including the determination of the substrate specificities and the product structures, as well as the detection of other lineages’ TGS activities.

In summary, a gene (*pHTGS*) for 1,6-β-transglucosylase involved in storage β-glucan synthesis was identified in the haptophyte *P*. *haptonemofera*. The soluble recombinant protein was demonstrated to have activity-catalyzing glucan elongation using UDP-glucose as a substrate. This soluble enzyme system could be used to produce various structures of β-glucans, including biologically active glucans (Karuppusamy et al., 2022), as well as for basic studies on molecular mechanisms and evolution.

## Data Availability

The datasets presented in this study can be found in online repositories. The names of the repository/repositories and accession number(s) can be found at: DDBJ Repository (http://getentry.ddbj.nig.ac.jp/top-e.html)—accession number: LC773529.
