# Supplementary figures and images for: Kre6 (yeast 1,6-β-transglycosylase) homolog, PhTGS, is essential for β-glucan synthesis in the haptophyte Pleurochrysis haptonemofera
Source: Front Bioeng Biotechnol. 2023 Sep 18;11:1259587. doi: 10.3389/fbioe.2023.1259587 (PMC10543733; doi:10.3389/fbioe.2023.1259587)

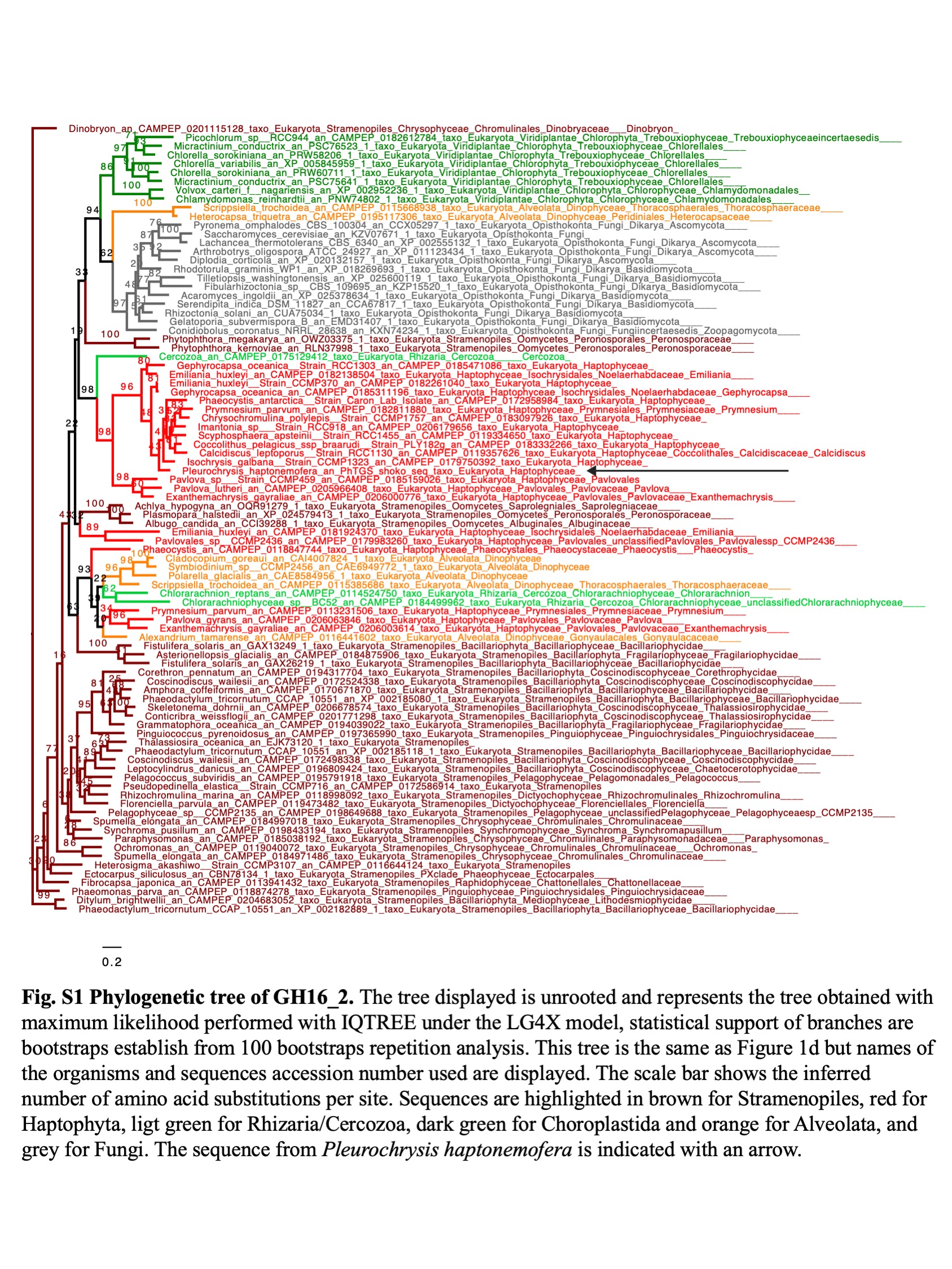

Supplement: Supplementary file 1 [file Image1.JPEG]
